# Supplementary material for: Opposing roles for TGFβ- and BMP-signaling during nascent alveolar differentiation in the developing human lung
Source: NPJ Regen Med. 2023 Sep 9;8:48. doi: 10.1038/s41536-023-00325-z (PMC10492838; doi:10.1038/s41536-023-00325-z)
Supplement: Supplementary file 1 — Supplementary Figures Legends and Tables [file 41536_2023_325_MOESM1_ESM.pdf]

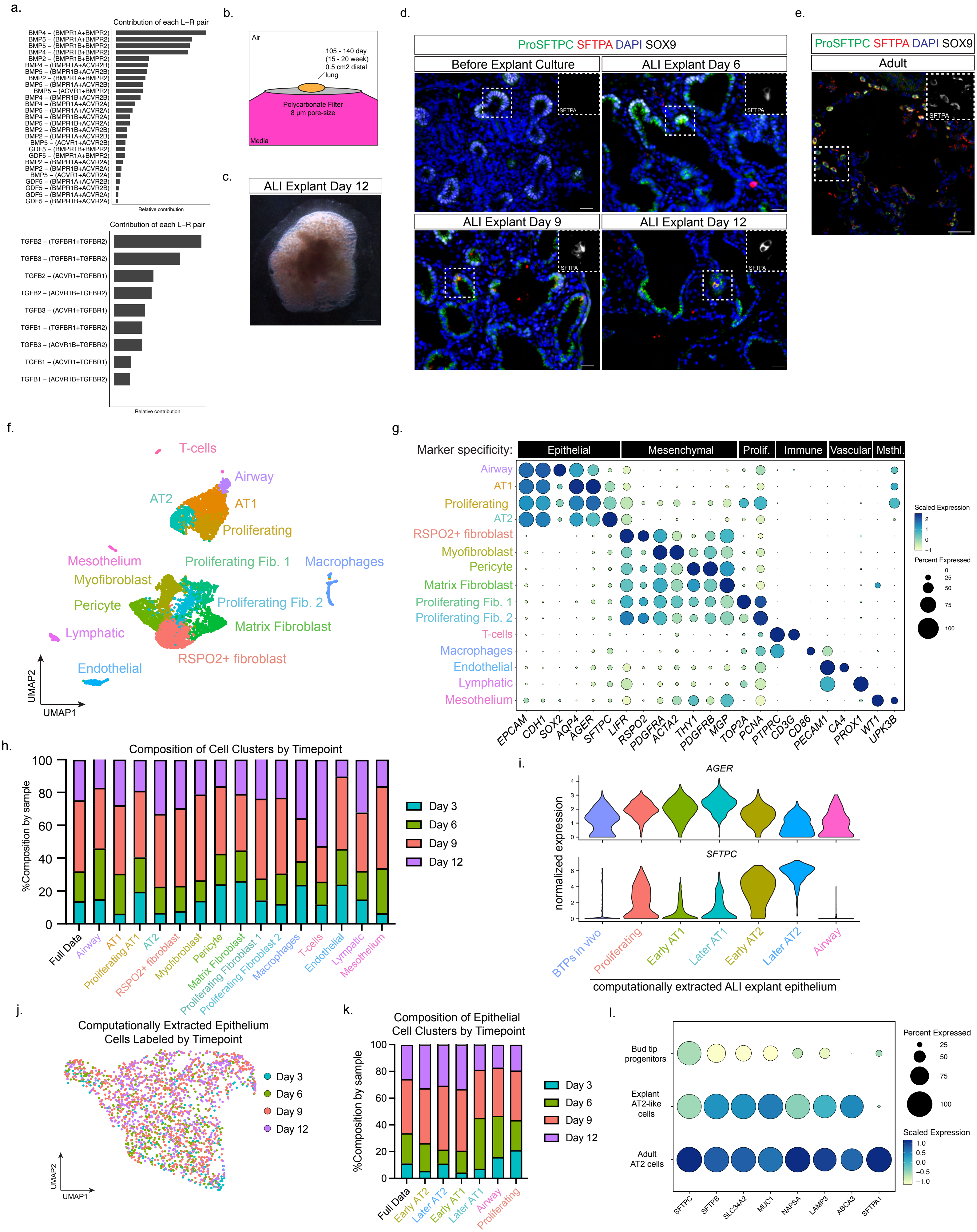
a.
Contribution of each L–R pair
BMP4 – (BMPR1A+BMPR2)
BMP5 – (BMPR1A+BMPR2)
BMP5 – (BMPR1B+BMPR2)
BMP4 – (BMPR1B+BMPR2)
BMP2 – (BMPR1B+BMPR2)
BMP4 – (BMPR1A+ACVR2B)
BMP5 – (BMPR1B+ACVR2B)
BMP2 – (BMPR1A+BMPR2)
BMP5 – (BMPR1A+ACVR2B)
BMP5 – (ACVR1+BMPR2)
BMP4 – (BMPR1B+ACVR2B)
BMP4 – (BMPR1A+ACVR2A)
BMP5 – (BMPR1A+ACVR2A)
BMP4 – (BMPR1B+ACVR2A)
BMP5 – (BMPR1B+ACVR2A)
BMP2 – (BMPR1B+ACVR2B)
BMP2 – (BMPR1A+ACVR2B)
BMP5 – (ACVR1+ACVR2B)
GDF5 – (BMPR1B+BMPR2)
GDF5 – (BMPR1A+BMPR2)
BMP2 – (BMPR1A+ACVR2A)
BMP2 – (BMPR1B+ACVR2A)
BMP5 – (ACVR1+ACVR2A)
GDF5 – (BMPR1A+ACVR2B)
GDF5 – (BMPR1B+ACVR2B)
GDF5 – (BMPR1A+ACVR2A)
GDF5 – (BMPR1B+ACVR2A)
Relative contribution
Contribution of each L–R pair
TGFB2 – (TGFBR1+TGFBR2)
TGFB3 – (TGFBR1+TGFBR2)
TGFB2 – (ACVR1+TGFBR1)
TGFB2 – (ACVR1B+TGFBR2)
TGFB3 – (ACVR1+TGFBR1)
TGFB1 – (TGFBR1+TGFBR2)
TGFB3 – (ACVR1B+TGFBR2)
TGFB1 – (ACVR1+TGFBR1)
TGFB1 – (ACVR1B+TGFBR2)
Relative contribution
b.
Air
105 - 140 day
(15 - 20 week)
0.5 cm2 distal
lung
Polycarbonate Filter
8 µm pore-size
Media
c.
ALI Explant Day 12
d.
ProSFTPC SFTPA DAPI SOX9
Before Explant Culture
ALI Explant Day 6
ALI Explant Day 9
ALI Explant Day 12
SFTPA
e.
ProSFTPC SFTPA DAPI SOX9
Adult
SFTPA
f.
T-cells
Airway
AT2
AT1
Proliferating
Mesothelium
Proliferating Fib. 1
Macrophages
Myofibroblast
Pericyte
Proliferating Fib. 2
Lymphatic
Matrix Fibroblast
Endothelial
RSPO2+ fibroblast
UMAP2
UMAP1
g.
Marker specificity:
Epithelial
Mesenchymal
Prolif.
Immune
Vascular
Msthl.
Airway
AT1
Proliferating
AT2
RSPO2+ fibroblast
Myofibroblast
Pericyte
Matrix Fibroblast
Proliferating Fib. 1
Proliferating Fib. 2
T-cells
Macrophages
Endothelial
Lymphatic
Mesothelium
EPCAM CDH1 SOX2 AQP4 AGER SFTPC LIFR RSPO2 PDGFRA ACTA2 THY1 PDGFRB MGP TOP2A PCNA PTPRC CD3G CD86 PECAM1 CA4 PROX1 WT1 UPK3B
Scaled Expression
2
1
0
−1
Percent Expressed
0
25
50
75
100
h.
Composition of Cell Clusters by Timepoint
%Composition by sample
100
80
60
40
20
0
Day 3
Day 6
Day 9
Day 12
Full Data
Airway
AT1
Proliferating AT1
AT2
RSPO2+ fibroblast
Myofibroblast
Pericyte
Matrix Fibroblast
Proliferating Fibroblast 1
Proliferating Fibroblast 2
Macrophages
T-cells
Endothelial
Lympatic
Mesothelium
i.
AGER
SFTPC
normalized expression
BTPs in vivo
Proliferating
Early AT1
Later AT1
Early AT2
Later AT2
Airway
computationally extracted ALI explant epithelium
j.
Computationally Extracted Epithelium
Cells Labeled by Timepoint
Day 3
Day 6
Day 9
Day 12
UMAP2
UMAP1
k.
Composition of Epithelial
Cell Clusters by Timepoint
%Composition by sample
100
80
60
40
20
0
Day 3
Day 6
Day 9
Day 12
Full Data
Early AT2
Later AT2
Early AT1
Later AT1
Airway
Proliferating
l.
Bud tip
progenitors
Explant
AT2-like
cells
Adult
AT2 cells
SFTPC SFTPB SLC34A2 MUC1 NAPSA LAMP3 ABCA3 SFTPA1
Percent Expressed
25
50
75
100
Scaled Expression
1.0
0.5
0.0
−0.5
−1.0

Supplementary Fig. 1: Ligand-receptor pairs contributing to BMP- and TGFβ-signaling during fetal lung development and characterization of canalicular stage lung air-liquid interface (ALI) explant culture. Related to Figure 1.

a) Ligand receptor pairs contributing to cell-cell signaling predictions in Figure 1a.

b) Schematic of lung explant air-liquid interface culture. 0.5 cm^2 pieces of distal canalicular stage lung are cultured on polycarbonate filters that float on growth-factor and serum-free media.

c) Bright-field image of explant ALI cultures at day 12. Media is observed around the base of the explant, but otherwise the explant is exposed to air. Scale = 1mm.

d, e) Immunofluorescent staining of AT2 markers (ProSFTPC, SFTPA) and BTP marker SOX9 in (d) canalicular stage lung explants before and after 6, 9 or 12 days of explant ALI culture or (e) adult lung. Scale = 25μm.

f) UMAP visualization of Louvain clustering of all cells from day 3, day 6, day 9 and day 12 explants. Cluster identities were assigned based on marker expression in part g.

g) Dot plot showing cluster specific marker expression. Marker specificity on the top row denotes cell type/state indicated by unique expression of markers shown. Prolif. = proliferation. Msthl. = mesothelium.

h) Quantification of the percent contribution of each timepoint to clusters identified in integrated scRNA-sequencing data from ALI explant culture. The contribution of each timepoint to the full dataset is shown in the leftmost column.

i) Comparison of *AGER* (top) and *SFTPC* (bottom) expression between BTPs in lung tissue prior to ALI explant culture, and clusters identified in computationally extracted epithelial cells.

j) UMAP of computationally extracted epithelial cells from scRNA-sequencing of ALI explant culture with cells colored by days of ALI explant culture.

k) Percent contribution of each timepoint to clusters identified in computationally extracted

epithelium from integrated ALI explant scRNA-seq. The contribution of each timepoint to the full dataset is shown in the leftmost column.

I) Dot plot comparing expression of AT2 markers in BTPs, explant AT2-like cells and primary AT2 cells. Explant AT2-like cells express higher AT2 markers than bud tip progenitors and less than adult AT2 cells.

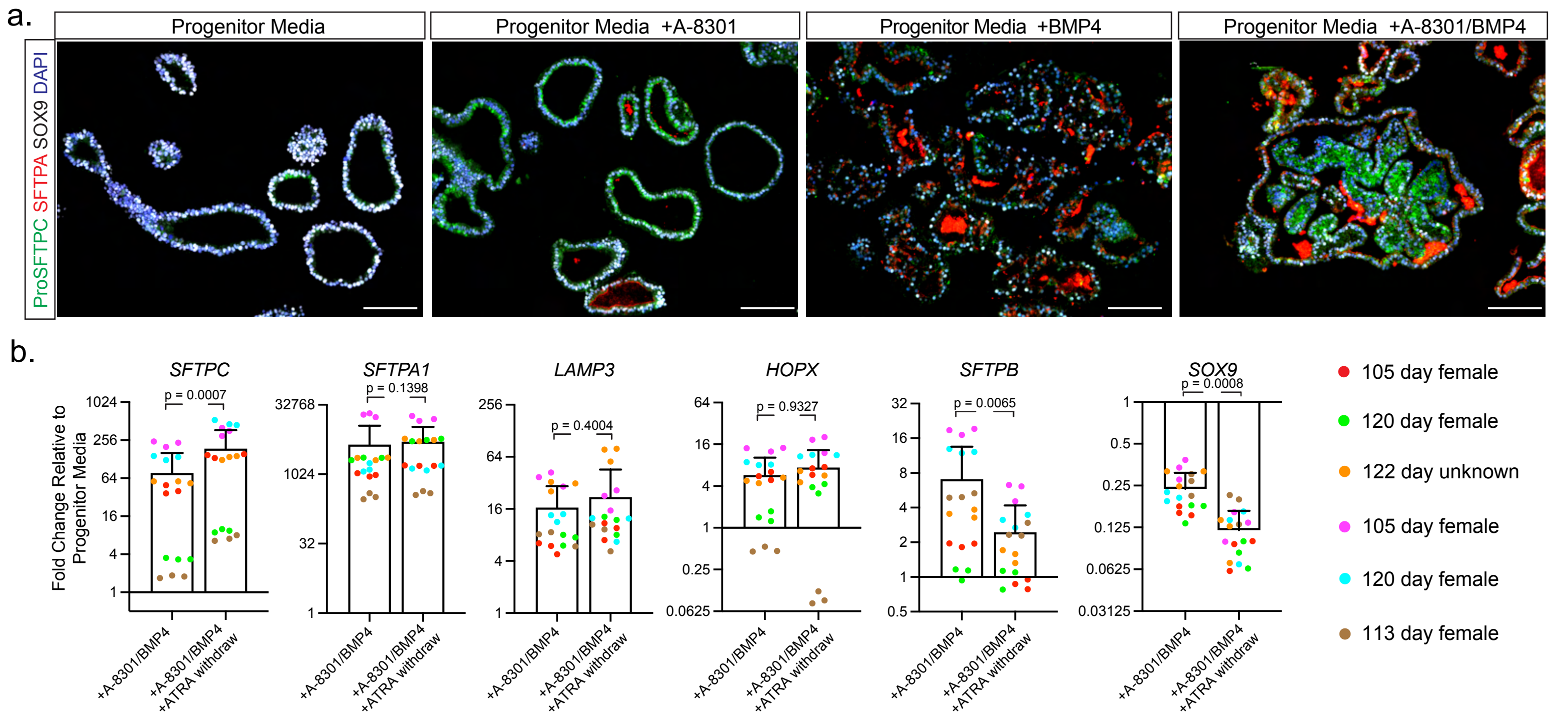
a.
Progenitor Media
Progenitor Media +A-8301
Progenitor Media +BMP4
Progenitor Media +A-8301/BMP4
ProSFTPC SFTPA SOX9 DAPI
b.
SFTPC
SFTPA1
LAMP3
HOPX
SFTPB
SOX9
Fold Change Relative to Progenitor Media
p = 0.0007
p = 0.1398
p = 0.4004
p = 0.9327
p = 0.0065
p = 0.0008
+A-8301/BMP4
+A-8301/BMP4 +ATRA withdraw
105 day female
120 day female
122 day unknown
105 day female
120 day female
113 day female

Supplementary Fig. 2: AT2 and BTP marker expression in BTP organoids under TGFβ-inhibition and BMP-activation and the effect of all-trans retinoic acid (ATRA) on AT2 differentiation of BTP organoids. Related to Figure 2.

a) Immunofluorescent staining images of AT2 markers (ProSFTPC and SFTPA) and BTP marker SOX9 in BTP organoids cultured in progenitor media or progenitor media with addition of TGFβ inhibitor A-8301 and BMP activator BMP4 alone or simultaneously for seven days. Scale = 100μm.

b) RT-qPCR measurements comparing AT2 markers (*SFTPC, SFTPA1, LAMP3, HOPX*, *SFTPB)* or BTP marker *SOX9* in response to simultaneous TGFβ-inhibition and BMP-activation in the presence (+A-8301/BMP4) or absence (+A8301/BMP4 +ATRA Withdraw) of ATRA for seven days. Values shown are fold change relative to BTP organoids maintained in Progenitor media (column = mean, error = s.d.). Statistical comparison (p) was computed by two-tailed ratio paired t-test on the mean arbitrary units of expression for six biological replicates calculated from three technical replicates.

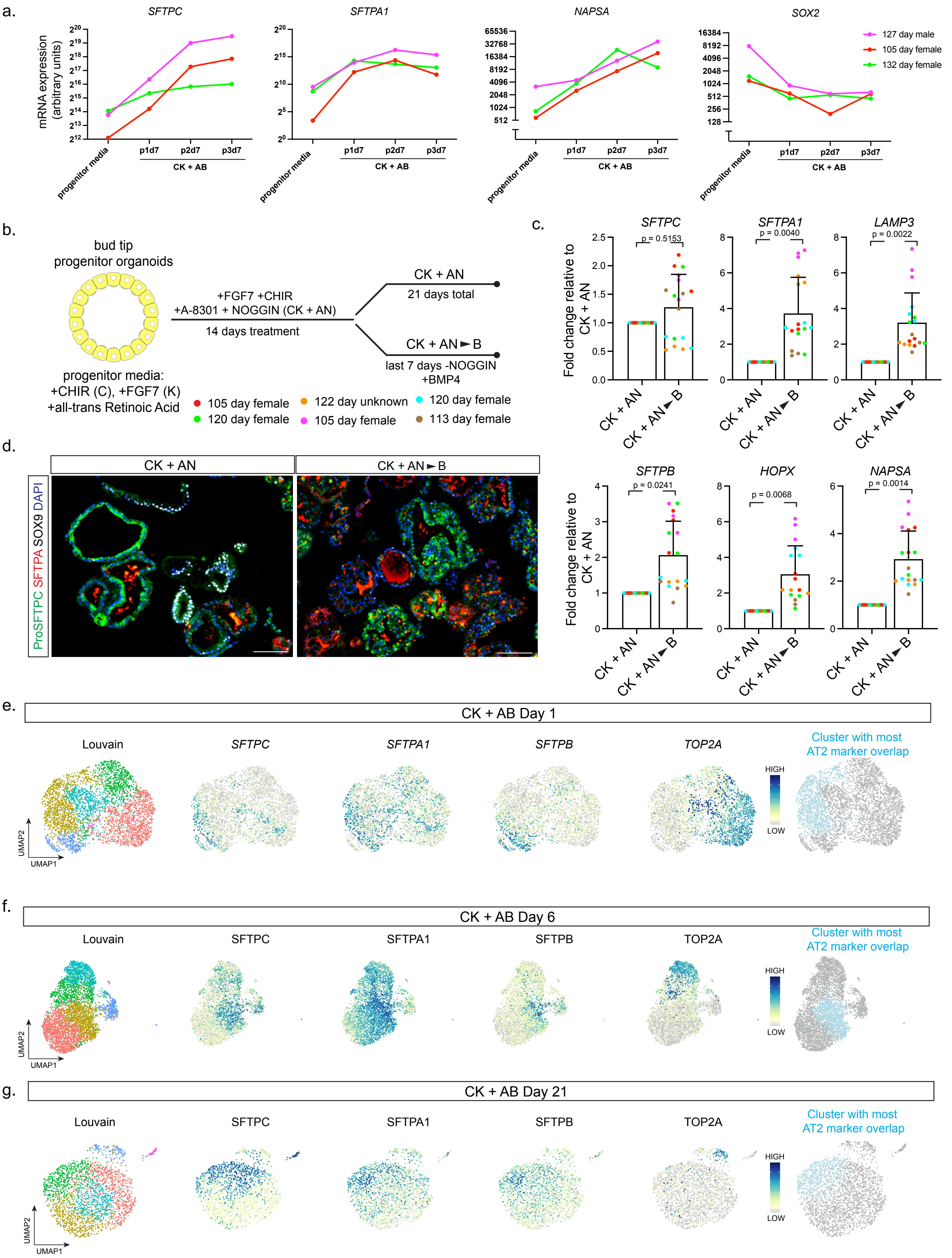
a.
SFTPC
SFTPA1
NAPSA
SOX2
mRNA expression (arbitrary units)
progenitor media
p1d7
p2d7
p3d7
CK + AB
127 day male
105 day female
132 day female
b.
bud tip progenitor organoids
progenitor media: +CHIR (C), +FGF7 (K) +all-trans Retinoic Acid
+FGF7 +CHIR +A-8301 + NOGGIN (CK + AN)
14 days treatment
CK + AN
21 days total
CK + AN ► B
last 7 days -NOGGIN +BMP4
105 day female
120 day female
122 day unknown
105 day female
120 day female
113 day female
c.
SFTPC
SFTPA1
LAMP3
SFTPB
HOPX
NAPSA
Fold change relative to CK + AN
p = 0.5153
p = 0.0040
p = 0.0022
p = 0.0241
p = 0.0068
p = 0.0014
CK + AN
CK + AN ► B
d.
CK + AN
CK + AN ► B
ProSFTPC SFTPA SOX9 DAPI
e.
CK + AB Day 1
f.
CK + AB Day 6
g.
CK + AB Day 21
Louvain
SFTPC
SFTPA1
SFTPB
TOP2A
HIGH
LOW
Cluster with most AT2 marker overlap
UMAP1
UMAP2

Supplementary Fig. 3: Reproducibility of CK + AB response in multiple BTP organoid lines, evidence for maximal differentiation in the presence of BMP-activation and identification of clusters with the most AT2 marker overlap at each CK + AB treatment timepoint. Related to Figure 3.

a) RT-qPCR measurements showing arbitrary units of expression for AT2 markers (*SFTPC, SFTPA1, NAPSA*) and airway marker *SOX2* in response to CK + AB over the course of 21 days for three BTP organoid lines. Values shown are mean arbitrary units of expression calculated from three technical replicates.

b) Schematic of BTP organoid differentiation experiment analyzed by RT-qPCR in part c and immunofluorescent staining in part d. Modified CK + AB media made to inhibit BMP-signaling rather than activate it by replacing BMP4 with NOGGIN (CK + AN) was applied to BTP organoids for 14 days. Cultures were then divided with half receiving CK + AB and the other half maintained in CK + AN with analysis performed after an additional 7 days (21 days total).

c) RT-qPCR measurements of AT2 marker expression in BTP organoids treated as schematized in part b. Values shown are fold change relative to the CK + AN condition color-coded by biological replicate (column = mean, error = s.d.). Statistical comparison (p) was computed by two-tailed ratio paired t-test on the mean arbitrary units of expression for six biological replicates calculated from three technical replicates.

d) Immunofluorescent staining for AT2 markers (ProSFTPC, SFTPA) and BTP marker SOX9 in BTP organoids differentiated under the conditions schematized in part b. Scale = 100µm.

e, f, g) UMAP visualization of Louvain clustering and gene expression for AT2 markers (*SFTPC, SFTPA1, SFTPB)* and proliferation marker *TOP2A* in CK + AB treated BTP organoids after (e) 1 day (f) 6 days (g) 21 days. The cluster with the highest overlapping expression of *SFTPC*, *SFTPA* and *SFTPB* is highlighted in the rightmost plot.

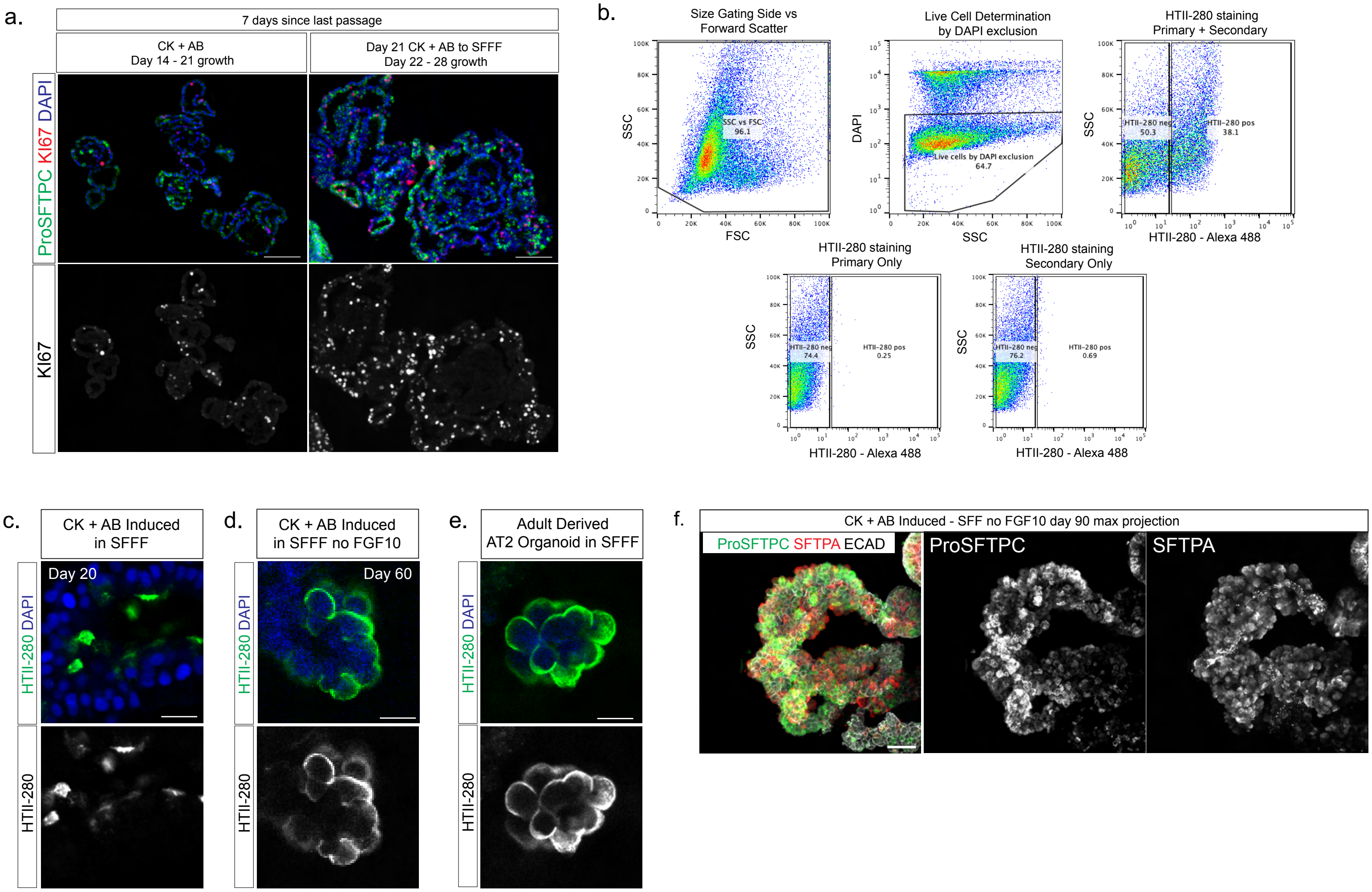

a.
7 days since last passage
CK + AB
Day 14 - 21 growth
Day 21 CK + AB to SFFF
Day 22 - 28 growth
ProSFTPC KI67 DAPI
KI67
b.
Size Gating Side vs
Forward Scatter
SSC vs FSC
96.1
SSC
FSC
Live Cell Determination
by DAPI exclusion
Live cells by DAPI exclusion
64.7
DAPI
SSC
HTII-280 staining
Primary + Secondary
HTII-280 neg
50.3
HTII-280 pos
38.1
HTII-280 - Alexa 488
HTII-280 staining
Primary Only
HTII-280 neg
74.4
HTII-280 pos
0.25
HTII-280 staining
Secondary Only
HTII-280 neg
76.2
HTII-280 pos
0.69
c.
CK + AB Induced
in SFFF
Day 20
HTII-280 DAPI
HTII-280
d.
CK + AB Induced
in SFFF no FGF10
Day 60
e.
Adult Derived
AT2 Organoid in SFFF
f.
CK + AB Induced - SFF no FGF10 day 90 max projection
ProSFTPC SFTPA ECAD
ProSFTPC
SFTPA

Supplementary Fig. 4: Proliferative and morphological features of CK + AB induced AT2-like organoids in CK + AB, SFFF and SFFF without FGF10 medias. Related to Fig. 4.

a) Immunofluorescent staining images of AT2 marker ProSFTPC and proliferation marker KI67 in CK + AB induced AT2-like organoids at the end of the final 7 days of 21 day CK + AB differentiation, or after the first 7 days of switching the culture to SFFF. Scale = 100µm.

b) FACS gating strategy to determine percent of cells expressing HTII-280 in Fig. 4g.

c,d,e) Immunofluorescent staining images of AT2 marker HTII-280 in CK + AB induced organoids expanded in SFFF for 20 days (c), CK + AB induced organoids expanded in SFFF without FGF10 for 60 days (d) or primary AT2 organoids in SFFF (e). Scales: (c) = 10µm, (d)(e) = 5µm.

f) Maximum signal intensity projection of confocal images from whole mount immunofluorescent staining of AT2 markers ProSFTPC and SFTPA in CK + AB induced organoids expanded in SFFF without FGF10 for 90 days. ECAD staining is included in the merged image to identify cell boundaries. Scale = 50µm.

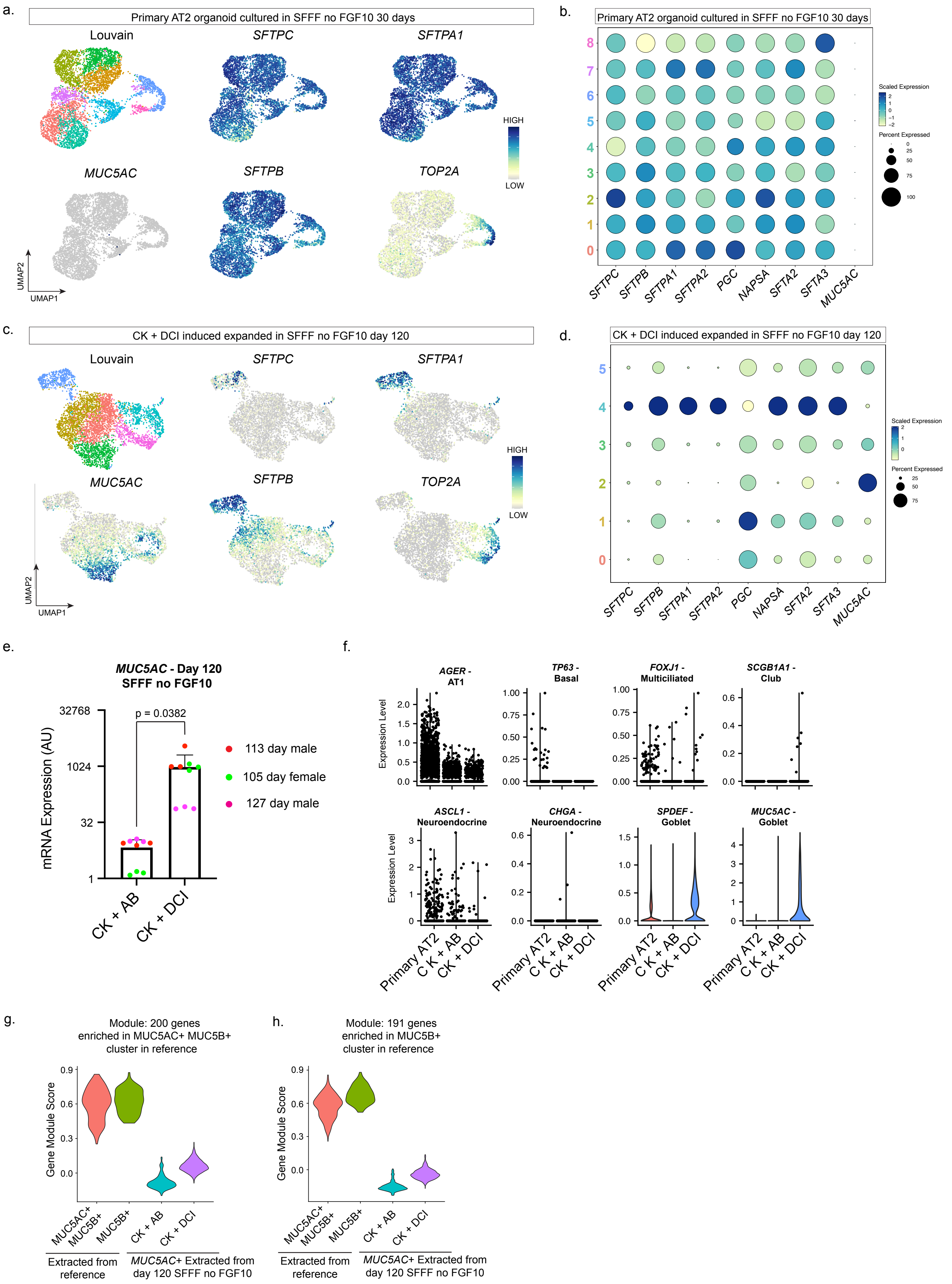
a.
Primary AT2 organoid cultured in SFFF no FGF10 30 days
Louvain
SFTPC
SFTPA1
MUC5AC
SFTPB
TOP2A
HIGH
LOW
UMAP2
UMAP1
b.
Primary AT2 organoid cultured in SFFF no FGF10 30 days
8
7
6
5
4
3
2
1
0
SFTPC
SFTPB
SFTPA1
SFTPA2
PGC
NAPSA
SFTA2
SFTA3
MUC5AC
Scaled Expression
Percent Expressed
c.
CK + DCI induced expanded in SFFF no FGF10 day 120
Louvain
SFTPC
SFTPA1
MUC5AC
SFTPB
TOP2A
HIGH
LOW
UMAP2
UMAP1
d.
CK + DCI induced expanded in SFFF no FGF10 day 120
5
4
3
2
1
0
SFTPC
SFTPB
SFTPA1
SFTPA2
PGC
NAPSA
SFTA2
SFTA3
MUC5AC
Scaled Expression
Percent Expressed
e.
MUC5AC - Day 120
SFFF no FGF10
32768
1024
32
1
p = 0.0382
mRNA Expression (AU)
CK + AB
CK + DCI
113 day male
105 day female
127 day male
f.
AGER - AT1
TP63 - Basal
FOXJ1 - Multiciliated
SCGB1A1 - Club
ASCL1 - Neuroendocrine
CHGA - Neuroendocrine
SPDEF - Goblet
MUC5AC - Goblet
Expression Level
Primary AT2
CK + AB
CK + DCI
g.
Module: 200 genes
enriched in MUC5AC+ MUC5B+
cluster in reference
Gene Module Score
MUC5AC+ MUC5B+
MUC5B+
CK + AB
CK + DCI
Extracted from reference
MUC5AC+ Extracted from day 120 SFFF no FGF10
h.
Module: 191 genes
enriched in MUC5B+
cluster in reference
Gene Module Score
MUC5AC+ MUC5B+
MUC5B+
CK + AB
CK + DCI
Extracted from reference
MUC5AC+ Extracted from day 120 SFFF no FGF10

Supplementary Fig. 5: Composition of primary AT2 and CK + DCI induced organoids expanded in primary AT2 media for 120 days and RT-qPCR validation of differences between expanded CK + AB and CK + DCI induced organoids. Related to Fig. 5.

a) UMAP visualization of Louvain clustering and gene expression in primary AT2 organoids cultured in SFFF without FGF10 for 30 days before analysis. AT2 markers (*SFTPC, SFTPA1, SFTPA*), proliferation marker *TOP2A* and goblet cell marker *MUC5AC* are shown.

b) Dot plot showing an expanded panel of AT2 markers and goblet cell marker MUC5AC across Louvain clusters in primary AT2 organoids cultured SFFF without FGF10 for 30 days before analysis.

c) UMAP visualization of Louvain clustering and gene expression in CK + DCI induced organoids expanded for 120 days in SFFF without FGF10. AT2 markers (*SFTPC, SFTPA1, SFTPB*), proliferation marker *TOP2A* and goblet cell marker *MUC5AC* are shown.

d) Dot plot showing expression of an expanded panel of AT2 markers and goblet cell marker *MUC5AC* in Louvain clusters in CK + DCI induced organoids expanded for 120 days in SFF without FGF10.

e) RT-qPCR measurements of *MUC5AC* expression in three BTP organoid lines induced with CK + AB or CK + DCI and expanded for 120 days in SFFF without FGF10. Values shown are arbitrary units of gene expression (column = mean, error = s.d.). Statistical significance (p) was calculated by one-tailed ratio paired t-test on the mean arbitrary units of expression for six biological replicates calculated from three technical replicates.

f) Violin plot comparing expression of markers of non-AT2 lung epithelial cell types in primary AT2 organoids.

g, h) Violin plots showing gene module scores for primary goblet cells extracted from an in vivo reference data set or goblet-like MUC5AC-positive cells extracted from day 120 CK + AB and CK + DCI AT2-like organoids in SFFF without FGF10. The gene module was comprised of the top (g) 200 genes enriched in the cluster annotated ‘MUC5AC+ MUC5B+’ in the in vivo

reference dataset or (h) 191 genes enriched in the cluster annotated ‘MUC5B+’ in the in vivo reference dataset.

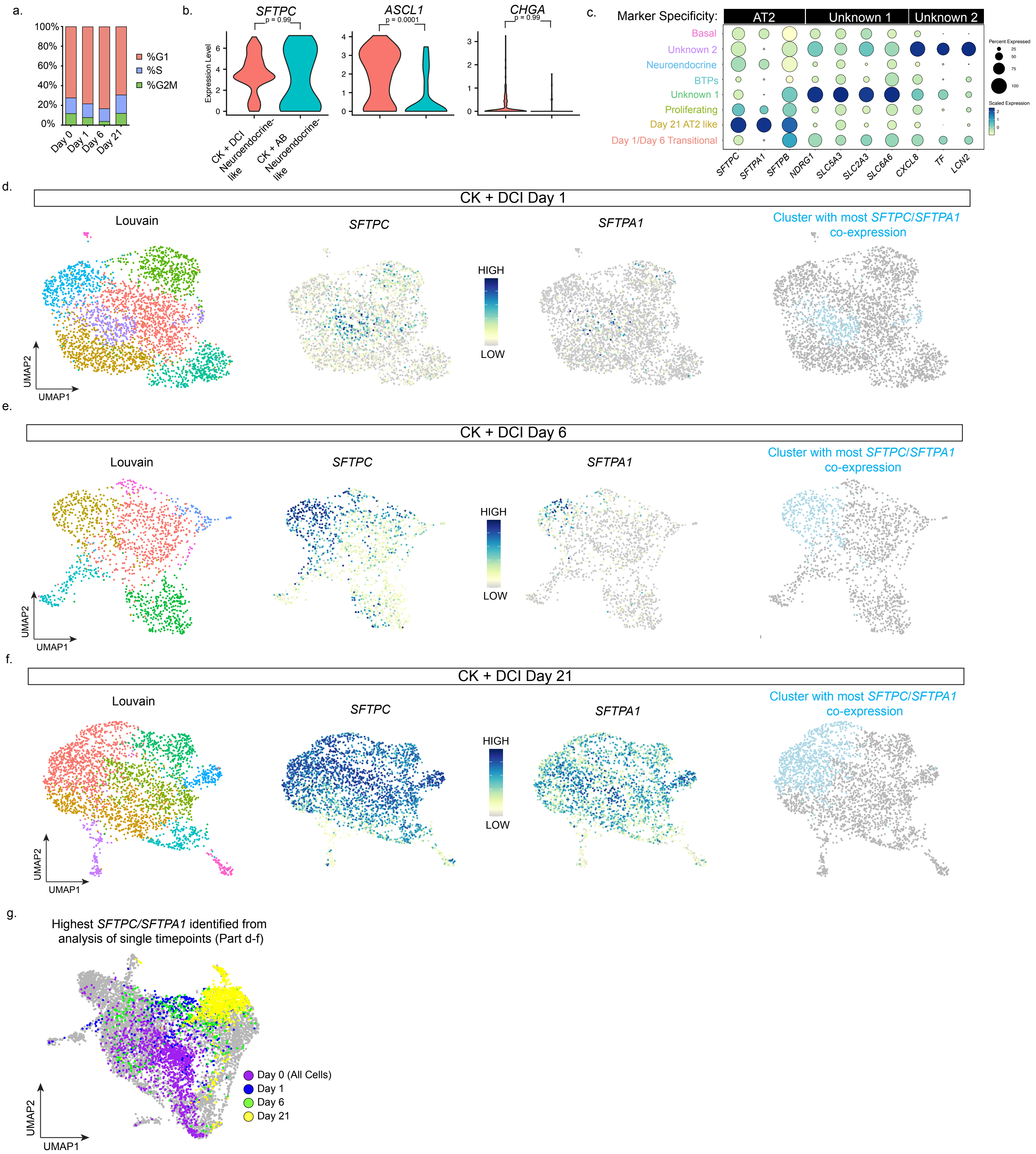
a.
100%
80%
60%
40%
20%
0%
Day 0
Day 1
Day 6
Day 21
%G1
%S
%G2M
b.
SFTPC
p = 0.99
ASCL1
p = 0.0001
CHGA
p = 0.99
Expression Level
CK + DCI Neuroendocrine-like
CK + AB Neuroendocrine-like
c.
Marker Specificity:
AT2
Unknown 1
Unknown 2
Basal
Unknown 2
Neuroendocrine
BTPs
Unknown 1
Proliferating
Day 21 AT2 like
Day 1/Day 6 Transitional
SFTPC
SFTPA1
SFTPB
NDRG1
SLC5A3
SLC2A3
SLC6A6
CXCL8
TF
LCN2
Percent Expressed
Scaled Expression
d.
CK + DCI Day 1
Louvain
SFTPC
SFTPA1
Cluster with most SFTPC/SFTPA1 co-expression
HIGH
LOW
UMAP2
UMAP1
e.
CK + DCI Day 6
Louvain
SFTPC
SFTPA1
Cluster with most SFTPC/SFTPA1 co-expression
HIGH
LOW
UMAP2
UMAP1
f.
CK + DCI Day 21
Louvain
SFTPC
SFTPA1
Cluster with most SFTPC/SFTPA1 co-expression
HIGH
LOW
UMAP2
UMAP1
g.
Highest SFTPC/SFTPA1 identified from analysis of single timepoints (Part d-f)
Day 0 (All Cells)
Day 1
Day 6
Day 21
UMAP2
UMAP1

Supplementary Fig. 6: Characterization of the transcription response of BTP organoids to CK + DCI and identification of clusters with the most AT2 marker overlap at each timepoint of CK + DCI treatment. Related to Figure 6.

a) Comparison of the percentage of cells in each phase of the cell cycle in BTP organoids (day 0) and at indicated day of CK + DCI treatment.

b) Violin plots comparing AT2 marker *SFTPC* or neuroendocrine marker expression (*ASCL1, CHGA*) in neuroendocrine-like cells present in either CK + DCI or CK + AB treated BTP organoids. Significance of differential enrichment (p) between the ASCL1-positive Neuroendocrine-like clusters from CK + DCI and CK + AB differentiations was determined by Wilcoxon Rank Sum test with Bonferroni correction.

c) Dot plot showing markers enriched in clusters of unknown identity from integrated scRNA-seq data of CK + DCI treatment time course.

d, e, f) UMAP visualization of Louvain clustering and gene expression for AT2 markers (*SFTPC, SFTPA1*) in CK + DCI treated BTOs after (d) 1 day (e) 6 days (f) 21 days. The cluster with the highest overlapping expression of *SFTPC* and *SFTPA1* is highlighted in the rightmost plot.

g) UMAP visualization of integrated scRNA-seq data from BTP organoids (day 0) and day 1, 6 and 21 of CK + DCI treatment with highest *SFTPC/SFTPA1* co-expressing cells from independent analysis of each scRNA-seq timepoint (part d-f) highlighted and color coded by timepoint.

Supplementary Table 1: Primary and secondary antibodies used for immunofluorescent staining.

Name	Company	Catalog #	Lot #	Dilution	Antibody Registry ID
Primary Antibodies					
Goat anti-SOX9	R&D Systems	AF3075	WIL0421041 WIL0420102	1:500	AB_2194160
Rabbit anti-SOX9	Millipore	AB5545	3587116	1:500	AB_2239761
Goat anti-ECAD	R&D Systems	AF748	CYG0421041	1:500	AB_355568
Rabbit anti-ProSFTPC	Seven Hills Bioreagents	WRAB-9337	364 458	1:500	AB_2335890
Mouse anti-ABCA3	Seven Hills Bioreagents	WMAB-17G524	17H524	1:500	AB_577285
Mouse anti-AGER	Abcam	ab54741	GR3287227-6	1:250	AB_2242462
Rabbit anti-PDPN	Santa Cruz Biotechnology	sc-134482	A0810	1:500	AB_2162079
Rabbit anti-HOPX	Santa Cruz Biotechnology	sc-30216	K0813	1:250	AB_2120833
Mouse anti-SFTPA	Leica	NCL-L-SPA	6077744	1:200	AB_564143
Mouse anti-SFTPC	Santa Cruz Biotechnology	sc-518029	A1921	1:200	AB_2937075
Goat anti-TP63	R&D Systems	BAF1916	KQH0218011 KQH0220041	1:500	AB_2207173
Mouse anti-HTII-280	Terrace Biotechnology	TB-27AHT2-280	B27	1:100	AB_2832931
Mouse anti-NAPSIN A	Leica	NCL-L-NAPSIN A	6081912	1:500	AB_10555426
Mouse anti-MUC5AC	Abcam	ab79082	GR2598331	1:500	AB_1603327
Rabbit anti-KI67	ThermoFisher	RM-9106-S1	910652007F	1:500	AB_2341197
Rabbit anti-pSMAD1/5/8	Millipore	AB3848	2002707	1:250	AB_177439
Secondary Antibodies					
AffiniPure donkey anti-mouse IgG	Jackson ImmunoResearch	715-545-150		1:500	AB_2340846

AlexaFluor 488					
AffiniPure donkey anti-mouse IgG Cyanine3	Jackson ImmunoResearch	715-165-150		1:500	AB_2340813
AffiniPure donkey anti-mouse IgG AlexaFluor 647	Jackson ImmunoResearch	715-605-150		1:500	AB_2340862
AffiniPure donkey anti-rabbit IgG AlexaFluor 488	Jackson ImmunoResearch	711-545-152		1:500	AB_2313584
AffiniPure donkey anti-rabbit IgG Cyanine3	Jackson ImmunoResearch	711-165-152		1:500	AB_2307443
AffiniPure donkey anti-rabbit IgG AlexaFluor 647	Jackson ImmunoResearch	711-605-152		1:500	AB_2492288
AffiniPure donkey anti-goat IgG AlexaFluor 488	Jackson ImmunoResearch	705-545-147		1:500	AB_2336933
AffiniPure donkey anti-goat IgG Cyanine3	Jackson ImmunoResearch	705-165-147		1:500	AB_2307351
AffiniPure donkey anti-goat IgG AlexaFluor 647	Jackson ImmunoResearch	705-605-147		1:500	AB_2340437
AffiniPure donkey anti-mouse IgM	Jackson ImmunoResearch	715-545-140		1:500	AB_2340845

Supplementary Table 2. Primer sequences for RT-qPCR.

Target	Sequence
SCGB3A2	F: GGGGCTAAGGAAGTGTGTAAATG<br>R: CACCAAGTGTGATAGCGCCTC
SFTPC	F: AGCAAAGAGGTCCTGATGGA<br>R: CGATAAGAAGGCGTTTCAGG
SFTPA1	F: TGTCCTCCTGGAAATGATGG<br>R: GGCTTGGAGCTCCTCATCTA
SFTPB	F: GGGTGTGTGGGACCATGT<br>R: CAGCACTTTAAAGGACGGTGT
SOX2	F: CCATCATTGGAGCAGGAATC<br>R: GACCAGCGGTAAGATTTCCA
MUC5AC	F: GCACCAACGACAGGAAGGATGAG<br>R: CACGTTCCAGAGCCGGACAT
NAPSA	F: TTCCGGGGCCACACTGAT<br>R: GGTTCTCTCCATCCCCTCAG
HOPX	F: GCCTTTCCGAGGAGGAGAC<br>R: TCTGTGACGGATCTGCACTC
LAMP3	F: GTTCTAAACGGAAGCAGACTCT<br>R: CGTTGGGGTCGATGTTGAAG
GAPDH	F: CTCTGCTCCTCCTGTTCGAC<br>R: TTAAAAGCAGCCCTGGTGAC

Supplementary Table 3: 199 genes enriched in primary alveolar type 2 organoids relative to bud tip progenitor organoids.

Gene Symbol				
SFTPC	*ALPL*	*ARPC1B*	*CA2*	*ALDH2*
SFTPA1	*RASGRF1*	*C1orf116*	*MT-ND4*	*DGKD*
SFTPA2	*SLC22A31*	*FTH1*	*HIF1A*	*ZMAT3*
SFTPB	*S100A14*	*CSTB*	*TMEM238*	*CD9*
NAPSA	*HOPX*	*MT-ND3*	*UQCR10*	*GALNT10*
SPINK5	*SFTA1P*	*LPCAT1*	*MT-ATP6*	*MEG3*
SLC34A2	*PHLDA2*	*DRAM1*	*SCNN1A*	*IFI16*
SERPIND1	*CYB5A*	*ETV1*	*HHIP-AS1*	*CREG1*
HPGD	*CTSD*	*NFIC*	*STC1*	*HLA-A*
SCGB3A1	*LMO3*	*NNMT*	*CBR1*	*SELENBP1*
PIGR	*CD59*	*NQO1*	*MTUS1*	*DHCR24*
AQP1	*C19orf33*	*NPC2*	*ACSL4*	*ARHGDIB*
LRRK2	*SLC39A8*	*ITGB6*	*RAB27B*	*TST*
SFTA2	*C2*	*RAB27A*	*SNHG7*	*STOM*
HLA-DRA	*MT-CO2*	*HSPH1*	*LY6E*	*ACSL1*
HHIP	*CACNA2D2*	*EPHX1*	*MEGF9*	*SLC66A1L*
CEACAM6	*LGI3*	*LGALS3*	*CEBPA*	*CTSS*
CD74	*SDR16C5*	*MSMO1*	*SLC6A20*	*FAH*
AQP5	*HLA-DRB1*	*FBP1*	*SNX25*	*PARM1*
VEPH1	*MICAL2*	*GPX4*	*ANXA1*	*COX17*
NUPR1	*TFPI*	*BCL2L1*	*CYP1B1*	*TMEM125*
FTL	*TNC*	*NFIX*	*SDC1*	*HLA-DOA*
SFTPD	*S100A6*	*HLA-C*	*TGFBR2*	*MCUR1*
SERPINA1	*POLR2L*	*BRI3*	*ADIPOR1*	
MFSD2A	*KRT7*	*ICAM1*	*DCXR*	
HLA-B	*SNX30*	*SCD*	*CAPN2*	
LAMP3	*HIP1*	*HLA-DMA*	*MMP28*	
MT-ND4L	*SFRP5*	*C16orf89*	*TSTD1*	
ADGRF5	*ABCA3*	*FGGY*	*ACSS2*	
HLA-DPA1	*CAT*	*AK1*	*MT-CO1*	
HLA-DPB1	*MYO1B*	*FOLR1*	*GGTLC1*	
SLPI	*IL18*	*MSN*	*DCBLD2*	
S100A9	*MT-ND5*	*ISCU*	*TGM2*	
CD36	*MPZL2*	*HPCAL1*	*HSPB8*	
SUSD2	*CHI3L1*	*LIPH*	*CPM*	
DBI	*DUOX1*	*MGST1*	*PID1*	
XIST	*CDC25B*	*CAPN8*	*RHOBTB2*	
TMEM213	*SDC4*	*IL1R1*	*EPDR1*	
FASN	*AP000357.2*	*PLXND1*	*QPRT*	
ALOX15B	*SFN*	*MEGF6*	*SULT1A1*	
CTSH	*RPS26*	*MBNL1*	*ADGRF1*	
CCND2	*SELENOW*	*GGT5*	*TAOK3*	
C3	*B2M*	*PDXK*	*MID1IP1*	
MALL	*SELENOP*	*CXCL17*	*AQP4*	

Supplementary Table 4: 199 genes enriched in primary alveolar type 2 cells relative to all other lung cell types. List from reference 29.

Gene Symbol				
SFTPC	*TMEM163*	*MSMO1*	*PID1*	*HLA-DMB*
NPC2	*CXCL2*	*KIAA1324L*	*AQP1*	*CYP51A1*
SFTPA1	*SFTA3*	*SNX30*	*HSD17B4*	*CHP1*
NAPSA	*HHIP-AS1*	*ETV1*	*SNX25*	*CREB3L1*
SFTPA2	*CD74*	*PARM1*	*PEBP1*	*GSPT1*
CTSH	*ETV5*	*ZNF385B*	*FGG*	*CD83*
PGC	*SLC34A2*	*FASN*	*C1orf21*	*BRI3*
SFTPD	*DMBT1*	*FBP1*	*SPTSSA*	*HMGCS1*
LAMP3	*FGGY*	*HMOX1*	*IDI1*	*PTP4A3*
ABCA3	*HLA-DRB1*	*CITED2*	*CXCL17*	*SOCS3*
CHI3L2	*RASGRF1*	*PLD3*	*AKAP13*	*SERPINB1*
CA2	*NECAB1*	*PMM1*	*BTG1*	*AZGP1*
DBI	*SELENBP1*	*CDC42EP1*	*FMO5*	*PNRC1*
SERPINA1	*SDR16C5*	*ODC1*	*FDPS*	*SOD2*
WIF1	*TFPI*	*ORM1*	*RAB27A*	*XBP1*
LRRK2	*HOPX*	*HLA-DMA*	*TMSB4X*	*GADD45G*
C11orf96	*RND1*	*SPRY4*	*ASAH1*	*CSF3*
NRGN	*CD36*	*SMAGP*	*BLVRB*	*NFKBIZ*
SFTA2	*FABP5*	*ACADL*	*FLRT3*	*TXNIP*
PLA2G1B	*MUC1*	*B3GNT8*	*SECISBP2L*	*CXCL3*
HHIP	*RGS16*	*AGPAT2*	*CDK2AP2*	*PLIN2*
PEBP4	*ALPL*	*ESAM*	*RBPMS-AS1*	*SEC61G*
CPB2	*ALOX15B*	*ASRGL1*	*TSC22D1*	*MED24*
NNMT	*LRRC36*	*EPHX1*	*MRPL14*	
MFSD2A	*KCNJ15*	*LPL*	*CHCHD7*	
SFTPB	*CSF3R*	*QDPR*	*STC1*	
HLA-DPB1	*SCD*	*CISH*	*ADI1*	
CRTAC1	*LGALSL*	*MTRR*	*ATP6V0E1*	
C2	*LANCL1-AS1*	*CHI3L1*	*NTN4*	
MALL	*PPP1R1B*	*LGMN*	*LDHA*	
MID1IP1	*SLC46A2*	*CD44*	*TIFA*	
SLC22A31	*DCXR*	*HLA-DQB1*	*GEM*	
FTL	*C3*	*S100A14*	*SAT2*	
P3H2	*NFKBIA*	*MSN*	*STEAP4*	
HLA-DPA1	*BMP2*	*MLPH*	*SLC25A5*	
AK1	*DUSP6*	*GADD45B*	*TMEM41A*	
LHFPL3-AS2	*SLC6A14*	*MBIP*	*POLR2C*	
C4BPA	*HLA-DRB5*	*SOCS2*	*IFITM2*	
C16orf89	*AREG*	*GSTA4*	*LTA4H*	
CACNA2D2	*GKN2*	*EP300-AS1*	*TPD52L1*	
HLA-DRA	*CAT*	*TTN*	*ZFP36*	
TMEM243	*EDNRB*	*ACSL4*	*ENO1*	
DRAM1	*CEBPD*	*ZDHHC3*	*SCP2*	
LPCAT1	*KCNJ8*	*HP*	*CKS2*	
