# Supplementary material for: Opposing roles for TGFβ- and BMP-signaling during nascent alveolar differentiation in the developing human lung
Source: NPJ Regen Med. 2023 Sep 9;8:48. doi: 10.1038/s41536-023-00325-z (PMC10492838; doi:10.1038/s41536-023-00325-z)
Supplement: Supplementary file 2 — Reporting Summary [file 41536_2023_325_MOESM2_ESM.pdf]

nature portfolio

Corresponding author(s): Jason R. Spence

Last updated by author(s): Aug 11, 2023

Reporting Summary

Nature Portfolio wishes to improve the reproducibility of the work that we publish. This form provides structure for consistency and transparency in reporting. For further information on Nature Portfolio policies, see our Editorial Policies and the Editorial Policy Checklist.

Statistics

For all statistical analyses, confirm that the following items are present in the figure legend, table legend, main text, or Methods section.

n/a	Confirmed	
☐	☒	The exact sample size (*n*) for each experimental group/condition, given as a discrete number and unit of measurement
☐	☒	A statement on whether measurements were taken from distinct samples or whether the same sample was measured repeatedly
☐	☒	The statistical test(s) used AND whether they are one- or two-sided<br>*Only common tests should be described solely by name; describe more complex techniques in the Methods section.*
☐	☒	A description of all covariates tested
☐	☒	A description of any assumptions or corrections, such as tests of normality and adjustment for multiple comparisons
☐	☒	A full description of the statistical parameters including central tendency (e.g. means) or other basic estimates (e.g. regression coefficient) AND variation (e.g. standard deviation) or associated estimates of uncertainty (e.g. confidence intervals)
☐	☒	For null hypothesis testing, the test statistic (e.g. *F*, *t*, *r*) with confidence intervals, effect sizes, degrees of freedom and *P* value noted<br>*Give P values as exact values whenever suitable.*
☒	☐	For Bayesian analysis, information on the choice of priors and Markov chain Monte Carlo settings
☒	☐	For hierarchical and complex designs, identification of the appropriate level for tests and full reporting of outcomes
☒	☐	Estimates of effect sizes (e.g. Cohen's *d*, Pearson's *r*), indicating how they were calculated

Our web collection on statistics for biologists contains articles on many of the points above.

Software and code

Policy information about availability of computer code

Data collection	Single Cell Data was processed using CellRanger (10x Genomics)
Data analysis	Single Cell Data Analysis was performed using R packages Seurat, Slingshot and CellChat.<br>Custom Analysis Code used in the manuscript is available at https://github.com/jason-spence-lab/Frum-et-al.

For manuscripts utilizing custom algorithms or software that are central to the research but not yet described in published literature, software must be made available to editors and reviewers. We strongly encourage code deposition in a community repository (e.g. GitHub). See the Nature Portfolio guidelines for submitting code & software for further information.

Data

Policy information about availability of data

All manuscripts must include a data availability statement. This statement should provide the following information, where applicable:

- Accession codes, unique identifiers, or web links for publicly available datasets
- A description of any restrictions on data availability
- For clinical datasets or third party data, please ensure that the statement adheres to our policy

Single Cell Sequencing data used in this study is available at EMBL-EBI ArrayExpress, Gene Expression Omnibus or Synapse.org. EMBL-EBI ArrayExpress: Single-cell RNA sequencing of human fetal lung (E-MTAB-8221), human canancular stage lung ALI explants (E-MTAB-12959) (this study), and human lung organoids (E-

MTAB-12960) (this study). Gene Expression Omnibus: Single-cell RNA sequencing of micro-dissected human distal airways (GSE178360), Synapse.org: Human Lung Cell Atlas (syn21041850).

Research involving human participants, their data, or biological material

Policy information about studies with human participants or human data. See also policy information about sex, gender (identity/presentation), and sexual orientation and race, ethnicity and racism.

Reporting on sex and gender	Sex was reported but differences between sexes were not examined as it was not part of the original study design and sex-specific differences differences in means were not observed.
Reporting on race, ethnicity, or other socially relevant groupings	Socially relevant groupings were not collected.
Population characteristics	None known.
Recruitment	Samples were sent as available.
Ethics oversight	University of Michigan Institutional Review Board

Note that full information on the approval of the study protocol must also be provided in the manuscript.

Field-specific reporting

Please select the one below that is the best fit for your research. If you are not sure, read the appropriate sections before making your selection.

☒ Life sciences ☐ Behavioural & social sciences ☐ Ecological, evolutionary & environmental sciences

For a reference copy of the document with all sections, see nature.com/documents/nr-reporting-summary-flat.pdf

Life sciences study design

All studies must disclose on these points even when the disclosure is negative.

Sample size	Sample sizes were chosen based on the number of samples/lines available. We used n = 3 or 4 for experiments on fresh tissue and n = 4 or 6 for experiments on BTP organoid lines. For scRNA-sequencing data only n = 1 was performed. Conclusions from scRNA-sequencing were demonstrated to be reproducible across biological replicates by RT-qPCR.
Data exclusions	No data was excluded except for measurements of the 105 day female line in Fig. 4i measuring MUC5AC levels by qPCR. As noted in the text and methods this line lost AT2 markers but did not acquire MUC5AC expression in the presence of FGF10.
Replication	All replication experiments in this manuscript showed the findings to be reproducible across biological replicates.
Randomization	Samples were allocated as available. All experiments had treatments and controls paired by biological replicate.
Blinding	Blinding was not possible as experiments were collected and analyzed by the same researcher.

Reporting for specific materials, systems and methods

We require information from authors about some types of materials, experimental systems and methods used in many studies. Here, indicate whether each material, system or method listed is relevant to your study. If you are not sure if a list item applies to your research, read the appropriate section before selecting a response.

Materials & experimental systems

n/a	Involved in the study	
☐	☒	Antibodies
☐	☒	Eukaryotic cell lines
☒	☐	Palaeontology and archaeology
☒	☐	Animals and other organisms
☒	☐	Clinical data
☒	☐	Dual use research of concern
☒	☐	Plants

Methods

n/a	Involved in the study	
☒	☐	ChIP-seq
☐	☒	Flow cytometry
☒	☐	MRI-based neuroimaging

Antibodies

Antibodies used

Primary:
Goat anti-SOX9 R&D Systems AF3075 WIL0421041/WIL0420102
Rabbit anti-SOX9 Millipore AB5545 3587116
Goat anti-ECAD R&D Systems AF748 CYG0421041
Rabbit anti-ProSFTPC Seven Hills Bioreagents WRAB-9337 364/458
Mouse anti-ABCA3 Seven Hills Bioreagents WMAB-17G524 17H524
Mouse anti-AGER Abcam ab54741 GR3287227-6
Rabbit anti-PDPN Santa Cruz Biotechnology sc-134482 A0810
Rabbit anti-HOPX Santa Cruz Biotechnology sc-30216 K0813
Mouse anti-SFTPA Leica NCL-L-SPA 6077744
Mouse anti-SFTPC Santa Cruz Biotechnology sc-518029 A1921
Goat anti-TP63 R&D Systems BAF1916 KQH0218011/KQH0220041
Mouse anti-HTII-280 Terrace Biotechnology TB-27AHT2-280 B27
Mouse anti-NAPSIN A Leica NCL-L-NAPSIN A 6081912
Mouse anti-MUC5AC Abcam ab79082 GR2598331
Rabbit anti-KI67 ThermoFisher RM-9106-S1 910652007F
Rabbit anti-pSMAD1/5/8 Millipore AB3848 2002707

Secondary:
AffiniPure donkey anti-mouse IgG AlexaFluor 488 Jackson ImmunoResearch 715-545-150
AffiniPure donkey anti-mouse IgG Cyanine3 Jackson ImmunoResearch 715-165-150
AffiniPure donkey anti-mouse IgG AlexaFluor 647 Jackson ImmunoResearch 715-605-150
AffiniPure donkey anti-rabbit IgG AlexaFluor 488 Jackson ImmunoResearch 711-545-152
AffiniPure donkey anti-rabbit IgG Cyanine3 Jackson ImmunoResearch 711-165-152
AffiniPure donkey anti-rabbit IgG AlexaFluor 647 Jackson ImmunoResearch 711-605-152
AffiniPure donkey anti-goat IgG AlexaFluor 488 Jackson ImmunoResearch 705-545-147
AffiniPure donkey anti-goat IgG Cyanine3 Jackson ImmunoResearch 705-165-147
AffiniPure donkey anti-goat IgG AlexaFluor 647 Jackson ImmunoResearch 705-605-147
AffiniPure donkey anti-mouse IgM Jackson ImmunoResearch 715-545-140

Validation

Antibodies were validated to give the expected cell type specific staining pattern in human lung sections of the appropriate stage. The pSMAD1/5/8 antibody was validated by loss of nuclear signal upon BMP-inhibition with recombinant human NOGGIN.

Eukaryotic cell lines

Policy information about cell lines and Sex and Gender in Research

Cell line source(s)

Cell lines were derived from human fetal lung tissue.

Authentication

None of the cell lines were authenticated

Mycoplasma contamination

Cell lines were not tested for mycoplasma contamination.

Commonly misidentified lines
(See ICLAC register)

None

Flow Cytometry

Plots

Confirm that:

☒ The axis labels state the marker and fluorochrome used (e.g. CD4-FITC).

☒ The axis scales are clearly visible. Include numbers along axes only for bottom left plot of group (a 'group' is an analysis of identical markers).

☒ All plots are contour plots with outliers or pseudocolor plots.

☒ A numerical value for number of cells or percentage (with statistics) is provided.

Methodology

Sample preparation

Organoids were retrieved from Matrigel by mechanical dissociation with a P1000, washed 2x with 1 mL PBS and, resuspended with TrypLE and incubated at 37ºC until a single cell suspension was obtained with light pipetting (typically 10 minutes). Cells were washed 3x with 1 mL of FACS buffer consisting of PBS, 2% BSA and 10 µM Y-27632 (APExBIO, Cat#3008) and then filtered through a 30 µM mesh. Cells were stained for 1 hour on ice in FACS buffer with a 1:60 dilution of anti HTII-280 IgM antibody. Cells were washed 3x with 1 mL of FACS buffer before 30 minutes of staining in FACS buffer with a 1:1000 dilution of anti-mouse IgM-Alexaflour-488 secondary antibody (Jackson Immunoresearch, Cat#715545140) on ice. After 3x washes with 1 mL of FACS buffer cells were suspended in FACS buffer with 1:4000 dilution of DAPI.

Instrument	FACS was performed on either a Sony MA900 or the ThermoFisher Bigfoot Spectral Cell Sorter.
Software	FlowJo v10.
Cell population abundance	FACS was used to analyze the percent HTII-280-positive fraction of cells in AT2-like organoids in various medias. Sorted positive cells were not used for experiments in the manuscript.
Gating strategy	Cells were selected from debris on the basis of forward and side scatter. Live cells were selected based on DAPI exclusion. Live Cells were analyzed for HTII-280-488 and cells exhibiting higher fluorescence than primary-only and secondary-only controls were called as positive. Gating strategies were the same for each biological replicate and performed in the same session.<br>This is included in the Supplementary Information as Supplementary Fig. 4b.

☒ Tick this box to confirm that a figure exemplifying the gating strategy is provided in the Supplementary Information.
